# A five-year retrospective study of the epidemiological characteristics and visual outcomes of pediatric ocular trauma

**DOI:** 10.1186/s12886-018-0676-7

**Published:** 2018-01-18

**Authors:** Edita Puodžiuvienė, Giedrė Jokūbauskienė, Monika Vieversytė, Kirwan Asselineau

**Affiliations:** 1 Eye clinic, Lithuanian University of Health Sciences, Eivenių 2, Kaunas, Lithuania; 2 Ophthalmology department, Centre Hospitalier Universitaire de Limoges, 2, avenue Martin Luther King, Limoges, France

**Keywords:** Pediatric eye trauma, Ocular injury, Visual outcome

## Abstract

**Background:**

Pediatric trauma can lead to serious visual impairment as a result of the trauma itself or secondary to amblyopia. Precise data on epidemiological characteristics and visual outcomes of pediatric ocular injuries are valuable for the prevention of monocular blindness.

**Methods:**

A total of 268 cases of pediatric ocular trauma admitted to the Department of Ophthalmology of the Lithuanian University of Health Sciences Hospital from January 2008 to December 2013 were retrospectively reviewed. Data analysed included age, sex, cause, type and treatment of injury, initial and final visual acuity (VA) and tissues involvement. Eye injuries were classified by Birmingham Eye Trauma Terminology (BETT) and Ocular Trauma Classification System (OTCS).

**Results:**

The age of children ranged from 6 months to 17.5 years. Boys were more likely to suffer ocular injury than girls. Home was the leading place of eye injury (60.4%), followed by outdoors (31.7%), school (5.2%) and sporting area (2.2%). The highest percentage of eye injuries in children were caused by blunt (40.3%) and sharp objects (29.9%), followed by burns (9.3%), falls (6.7%), explosions (4.5%), fireworks (4.1%), gunshots (1.9%) and traffic accidents (0.7%). Closed globe injury (CGI) was the most common type of eye injury (53.4%). CGI were noted to be higher in children aged 13–18 years, while open globe injury (OGI) were higher in the pre-school age group. Injury of grade 4 and grade 5 were more common in OGI, while grade 1 and grade 2 predominated in cases of CGI. Hypotony, traumatic cataract, iris laceration, vitreous prolapse and uveitis were the most common presentations of OGI, while hyphema, secondary glaucoma and retinal edema were significantly related with CGI. Final diagnoses contributing to poor final visual outcome such as corneal scar corneal opacity, hypotony, aphakia, and retinal detachment were statistically significant related only with OGI. Overall, 65.63% of children regained good visual acuity (VA ≥ 0.5), but for 18.4% of them, the trauma resulted in severe visual impairment (VA ≤ 0.1).

**Conclusion:**

Ocular trauma in children still remains an important preventable cause of ocular morbidity. This study provides data indicating that ophthalmological injuries are a significant cause of visual impairment in children.

**Electronic supplementary material:**

The online version of this article (10.1186/s12886-018-0676-7) contains supplementary material, which is available to authorized users.

## Background

Ocular injuries are the most common cause of acquired monocular blindness in children [1, 2]. Yet they have not received the attention that they deserve [2]. The rate of hospitalization for pediatric eye injuries in the United States in 2000 was 8.9 per 100,000 persons aged 20 years or less [3]. Worldwide, the incidence of severe visual impairment or blindness, caused by ocular trauma in children varies from 2% to 14% according to different studies [4, 5]. Children are more susceptible to eye injuries due to immature motor skills and limited common sense, they have a natural curiosity and are often seen imitating with no regards to the risks and outcomes. Although most eye injuries are avoidable by simple preventive measures, many children suffer visual impairment that can affect their psychosocial development [6, 7]. The diagnosis and treatment of ocular injuries in children are particularly challenging as the primary assessment can be very difficult for uncooperative young patients. [4]. In addition, the postoperative course can be more complicated in children due to an increased ocular inflammatory response, along with the development of amblyopia and the poor compliance to the treatments [8]. The visual outcome of ocular trauma depends on many factors including the etiology, severity and most importantly the duration from the injury until the surgery [9].

The objective was to analyse and study the epidemiological aspects and outcomes of pediatric ocular injuries admitted to a tertiary ophthalmological centre in Lithuania over a five-year period. Pediatric ocular trauma can lead to severe morbidity as the result of visual impairment, therefore, the data gathered were analysed in order to identify and classify the trauma by etiology, outcome and factors influencing the prognosis. No such study has been yet performed and published in Lithuania, hence the necessity to fill a void regarding this population’s ocular trauma.

## Methods

This retrospective study adhered to the tenets of the Declaration of Helsinki and received the approval from the Regional Committee of Bioethics (N° BEC-MF-679). The medical histories of all pediatric patients with serious eye injuries admitted to the Department of Ophthalmology of the Lithuanian University of Health Sciences Hospital in Kaunas, Lithuania, from January 2008 to December 2013 were included and reviewed in the study, totalizing 268 cases. The follow up of all these patients took place in the outpatient department of the same Hospital, during which a complete ophthalmological examination was performed and the treatment adjusted. Several parameters were investigated from the medical records: demographic (gender, age), nature of injury (type, mechanism, cause, place), initial ophthalmological examination (VA, measured by Snellen or Landolt „C “chart for preverbal children, anterior segment biomicroscopy, fundus examination when possible, preliminary diagnosis), management (type and number of surgeries, time from injury to admission and time from injury to surgery), follow up dates and information obtained at the end of follow up period (final best corrected VA and final diagnoses). The population was then further divided into three groups according to the children’s age: < 7 years (pre-school), 7–12 years, 13–18 years old. The classification of eye injuries was based on the Birmingham Eye Trauma Terminology (BETT) [10] and the Ocular Trauma Classification System (OTCS) [11]. The types of OGI were classified as globe rupture, penetrating injury, intraocular foreign body injury and perforating injury, while CGI were identified as eye contusions. OGI were further classified according to the affected zone (as defined in the OTCS), and both types were classified according to the grade of injury, defined by the presenting VA: grade 1 (≥0.5), grade 2 (0.2–0.4), grade 3 (0.03–0.1), grade 4 (light perception, LP-0.02), grade 5 (no light perception, NLP).

The Statistical Package for Social Science (SPSS 22.0) was used for statistical analysis.

A statistical analysis of all quantitative data, including descriptive statistics, parametric and non-parametric comparisons was performed for all variables. Chi-square and Fischer’s exact test were performed to test differences in proportions of categorical variables between two or more groups, and the Wilcoxon nonparametric test was used for dependant variables. A *p* < 0.05 value was considered statistically significant.

## Results

**Fig. 1:**
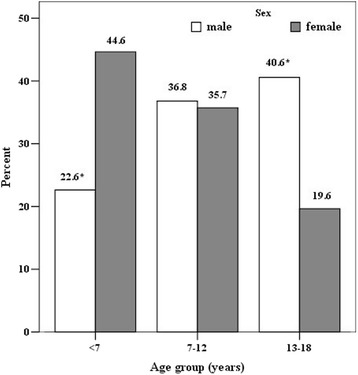
Distribution of trauma by sex and by age group (in %)

A total number of 268 pediatric ocular injury cases have been admitted to the hospital during the study period**.** The age of children ranged from 6 months to 17.5 years (10.7 ± 4.6 years). Among unilateral injuries, no significant difference was observed between the affected eye (right eye 49.3% vs. left eye 50.7%), 9.7% of all eye trauma were bilateral. The study population was divided into three age groups: < 7 years (27.2%, *n* = 73), 7–12 years (36.6%, *n* = 98) and 13–18 years (36.2%, *n* = 97) old. Boys were statistically more likely to experience ocular injury than girls (ratio 3.8:1; *p* < 0.001). As seen in the Fig. 1, eye trauma frequency increased considerably over age of 13 for boys and under 7 for girls.

Home was the leading place of eye injury (60.4%, *n* = 162), followed by outdoor environment (31.7%, *n* = 85), school (5.2%, *n* = 14) and sporting area (2.2%, *n* = 6). Table 1 provides information about the distribution of age and place of injury. Home was the most frequent place of injury in the pre-school age group, while outdoors activities and street in the age group over the age of 13 years (*p* < 0.05).

**Table 1:** Place of injury according to age

Place of injury	Age group (years)		
	< 7	7–12	13–18
	*n*(%)		
Home	64(88.9)^*,**^	56(57.1)^*^	42(43.3)^**^
School	1(1.4)	8(8.2)	5(5.2)
Outdoor activities	2(2.28)^*,**^	17(17.3)^*^	23(23.7)^**^
Street	5(6.9)^**^	15(15.3)	23(23.7)^**^
Sports	0	2(2.0)	4(4.1)

(^*,**^p < 0.01 between age groups)

The highest percentage of eye injuries in children were caused by blunt (40.3%, *n* = 108) and sharp objects (29.9%, *n* = 80), followed by burns (9.3%, *n* = 25), falls (6.7%, *n* = 18), explosions (4.5%, *n* = 12), fireworks (4.1%, *n* = 11), gunshots (1.9%, *n* = 5) and traffic accidents (0.7%, n = 2). Blunt objects, fireworks and burns accounted for a greater number of eye injuries in older children study groups (7–12 and 13–18 years), while sharp objects were a more common cause of injury in the pre-school age group (Table 2).

**Table 2:** Cause of injury according to age

Cause of injury	Age group (years)		
	< 7	7–12	13–18
	*n*(%)		
Sharp object	37(50.7)^*,**^	26(26.5)^*^	17(17.5)^**^
Blunt object	21(28.8)^*,**^	44(44.9)^*^	43(44.3) ^**^
Fall	5(6.8)	5(5.1)	8(8.2)
Gunshot	2(2.7)	1(1.0)	2(2.7)
Burn	6(8.2)	5(5.1)^***^	14(14.4)^***^
Explosion	0^*^	8(8.2)^*^	4(4.1)
Traffic accident	0	1(1.0)	1(1.0)
Fireworks	0^*,**^	5(5.1)^*^	6(6.2)^**^
Lawn equipment	1(1.4)	0	1(1.0)
Other	1(1.4)	3(3.0)	1(1.0)

(^*,**,***^p < 0.05 between age groups)

**Fig. 2:**
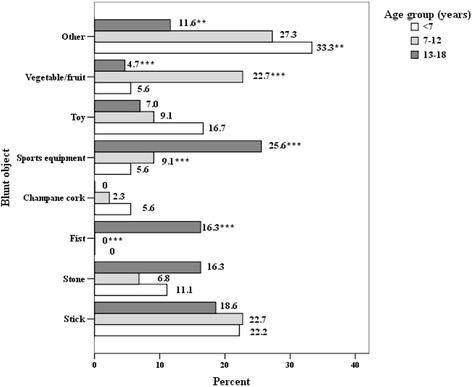
Distribution of blunt objects trauma by age (in %)

**Fig. 3:**
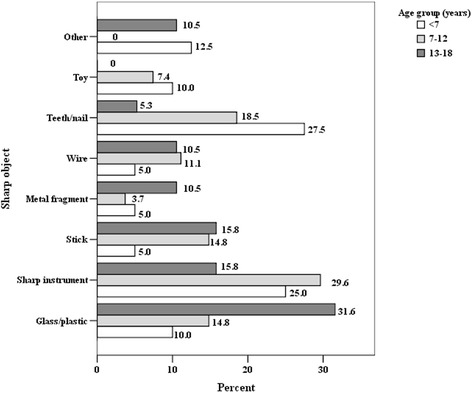
Distribution of sharp objects by age (in %)

The most common blunt object was a wooden stick (24.8%, *n* = 28) with no statistical differences between all study age groups, but sports equipment (14.2%, *n* = 16), stone (12.4%, *n* = 14) and fruit or vegetable (11.5%, *n* = 13) were significantly more frequent in children over the age of 12. The distribution of blunt objects trauma according to age is illustrated in Fig. 2. The most frequently reported sharp objects, in decreasing order of frequency, were sharp instruments, glass/plastic fragments and animal bite, especially in the pre-school age group. No significant differences were identified between age and type of object (Fig. 3).

**Fig. 4:**
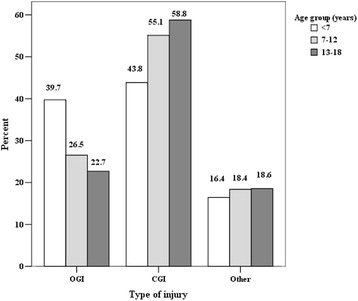
Type of injury by age (in %)

CGI were found to be the most common type of eye injury (53.4%; *n* = 77). OGI accounted for 28.7% (*n* = 143) overall, followed by ocular burns (9.3%) and non-globe injures such as eyelid lacerations, lacrimal system injury (8.6%). CGI were noted to be higher in children aged 13–18 years, and OGI – in the pre-school age group (*p* < 0.05) (Fig. 4).

The most frequent type of OGI were penetrating wounds (74%), followed by IOFB injury (22.1%), globe rupture (2.6%) and perforating wounds (1.3%), with no statistically significant differences when comparing the type of OGI and age. The injury location of zone I (wound limited to the corneal area, including the corneoscleral limbus) was noted in 73.7%, zone II (5 mm posterior to the corneoscleral limbus) in 15.8% and zone III (posterior to the anterior 5 mm of the cornea) in 10.5% of OGI. The distribution of IOFB location was as follows: 35.3% - magnetic IOFB in the posterior segment, 23.5% - nonmagnetic IOFB in the posterior segment, 23.5% nonmagnetic IOFB in the anterior segment and 11.8% – magnetic IOFB in the anterior segment.

The grade of injury, depending on the initial VA, in our study was as follows: grade 1–29.5%, grade 2–12.3%, grade 3–18.7%, grade 4–19.8%, grade 5–2.6%, not recorded/unknown – 17.2%. Table 3 presents the distribution of grade and type of injury. Grade 4 and grade 5 was mostly present in OGI, while grade 1 and grade 2 predominated in cases of CGI (*p* < 0.001).

**Table 3:** Grade of injury according to type

Grade (initial VA)	Type of injury	
	OGI	CGI
	*n*(%)	
1 (≥0.5)	12(15.6)	40(28.0)
2 (0.2–0.4)	3(3.9)	26(18.2)
3 (0.03–0.1)	9(11.7)	37(25.9)
4 (LP - 0.02)	29(37.7)	22(15.4)
5 (NLP)	5(6.5)	2(1.4)
Not recorded	19(24.7)	16(11.2)

Hypotony, traumatic cataract, iris laceration, vitreous prolapse and uveitis were the most common presentations of OGI, while hyphema, secondary glaucoma and retinal edema were the initial diagnoses significantly related with CGI (Table 4).

**Table 4:** Initial diagnoses by type of injury

Initial diagnosis	Type of injury		*p*
	OGI	CGI	
	*n*(%)		
Glaucoma, secondary	1(1.3)	19(13.3)	0.003
Hypotony	18(23.4)	6(4.2)	< 0.001
Hyphema	30(39.0)	103(72.0)	< 0.001
Lens: cataract	33(42.9)	9(6.3)	< 0.001
Lens: dislocation	3(3.9)	3(2.1)	0.435
Iris: prolapse	35(45.5)	0	< 0.001
Iris: dialysis	11(14.3)	9(6.3)	0.049
Iris:laceration	13(16.9)	5(3.5)	0.001
Vitreous: hemorrhage	18(23.4)	20(14.0)	0.079
Vitreous: prolapse	14(18.2)	3(1.4)	< 0.001
Retina: detachment	5(6.5)	1(0.7)	0.012
Retina: edema	8(10.4)	47(32.9)	< 0.001
Retina: hemorrhage	9(11.7)	29(20.3)	0.108
Retina: defect/tear	8(10.4)	12(8.4)	0.623
Choroid: rupture	2(2.6)	5(3.5)	0.717
Choroid: hemorrhage	3(3.9)	1(0.7)	0.124
Optic nerve injury	1(1.3)	2(1.4)	1.0
Orbital hemorrhage	3(3.9)	1(0.7)	0.124
Orbit: foreign body	3(3.9)	1(0.7)	0.124
Orbit: fracture	2(2.6)	0	0.121
Inflammation: uveitis	13(16.9)	5(3.5)	0.01
Inflammation: endophthalmitis	1(1.3)	0	0.35

Patients were admitted to the hospital on average 0.7 ± 1.6 days (range 0–14 days) after the injury: 64.9% during the first 24 h, 22.8% - in the first 45 h. Initial surgery was performed 1.53 ± 2.9 days after injury: 20.5% - during the first 24 h, 10.8% on the second day.

Overall, 152 surgeries were performed in 113 patients. An initial surgery was necessary in 113 pediatric patients (42.13%) and it was performed in 97.88% cases of OGI (wound closure 58.4%, lens removal 15.04%, IOFB removal 13.27%) and 23.98% of CGI (globe revision 8.85%, lens removal 1.77%, anterior chamber washout 0.88%, lacrimal system repair 4.42%). A second surgery was performed in 27.43% (31/113) of all the operated children, three surgeries in 7 cases and four surgeries for only one child. Additional surgeries were only performed in OGI.

The patients were then followed up in the outpatient department of the Hospital with a mean period of 327 ± 449 days (median 102 days). 

**Fig. 5:**
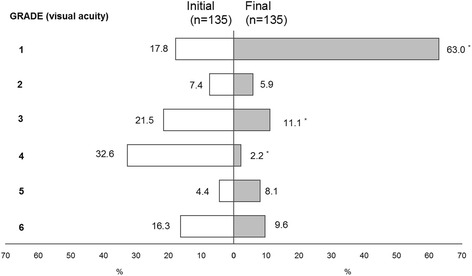
Relationship between initial and final visual acuity (in %)

The final VA was found in only 48.5% (130/268) of all patients’ record. Among them, 65.4% (85 eyes) regained a visual acuity of 0.5 or better. In 8 eyes (6.2%) final VA was 0.2–0.4, in 15 eyes (11.5%) 0.03–0.1, in 2 eyes (1.5%) - LP (HM), and in 7 eyes (5.4%)- NLP. Good visual outcome was significantly related with CGI, while VA of 0.03–0.1 and severe visual impairment (NLP) - with OGI (Table 5). 65.63% of all children regained good vision (VA ≥ 0.5), and 18.4% suffered severe visual impairment (VA ≤ 0.1). The relationship between initial and final BCVA is presented in Fig. 5.

**Table 5:** Final VA by type of injury

Grade (final VA)	Type of injury	
	OGI	CGI
	*n*(%)	
1^0^ (≥0.5)	27(44.3)^*^	49(81.7)
2^0^ (0.2–0.4)	4(6.5)	2(3.3)
3^0^ (0.03–0.1)	10(16.4)	5(8.3)
4^0^ (LP - 0.02)	3(4.9)	0
5^0^ (NLP)	10(16.4)^*^	1(1.7)
Not recorded	7(11.5)	3(5.0)

(^*^p < 0.05 between types of injury)

Final diagnoses such as corneal scar and opacity, iris deformity, hypotony, aphakia, posterior chamber IOL and retinal detachment, identified at the last follow up visit, were statistically significant related only with OGI (Table 6).

**Table 6:** Late diagnoses by type of injury

Late diagnosis	Type of injury		*p*
	OGI	CGI	
	*n*(%)		
Conjunctival scar	9(14.8)	3(5.0)	0.073
Corneal scar	52(85.2)	6(10.0)	< 0.001
Corneal opacity	38(62.3)	10(16.7)	< 0.001
Iris deformity	17(27.9)	8(13.3)	0.048
Glaucoma	5(8.2)	7(11.7)	0.523
Hypotony	9(14.8)	0	0.002
Iris atrophy	4(6.6)	3(5.0)	0.714
Cataract	13(21.3)	6(10.0)	0.087
Lens dislocation	2(3.3)	2(3.3)	0.987
Aphakia	10(16.4)	0	0.001
AC IOL	7(11.5)	3(5.0)	0.196
PC IOL	8(13.1)	1(1.7)	0.016
Vitreous hemorrhage	3(4.9)	2(3.3)	0.661
Retinal hemorrhage	2(3.3)	5(8.3)	0.234
Retinal edema	0	1(1.7)	0.311
Retinal defect	7(11.5)	12(20.0)	0.198
Retinal detachment	6(9.8)	0	0.013
PVR	1(1,6)	0	0.319
Macular degeneration	2(3.3)	3(5.0)	0.648
Retinal degeneration	3(5.0)	7(11.7)	0.186
Choroidal rupture	0	2(3.3)	0.15
Strabismus	9(15.0)	3(5.0)	0.073
Optic nerve avulsion	0	1(1.7)	0.311
Optic atrophy	1(1.7)	2(3.3)	0.549
Uveitis	1(1.7)	0	0.319
Orbital foreign body	0	1(1.7)	0.311
Anophthalmos	3(5.0)	0	0.082

## Discussion

Our study was motivated by the lack of data regarding pediatric ophthalmological trauma in Lithuania. Our aim was to obtain and summarize the epidemiological and clinical characteristics of severe ocular injuries in children, admitted to the Eye Clinic of the Lithuanian University of Health Sciences, the principal tertiary centre for ocular injuries in all the country.

Our study found that 72.8% of injuries occurred in children older than 7 years. Shoja et al. noted that a majority (58.3%) of injuries were seen in the 7–12 year of age group [12]. The higher incidence of ocular trauma in this age group is consistent with a study published by Sofi et al. [13] from Srinagar. Differing from our results, in their study conducted in Egypt, El-Sebaity et al. [1] found that the majority of pediatric trauma occurred in children aged 2–7 years of age. Similar results are also presented in other studies [5, 6, 14].

We identified a male gender predominance for ocular trauma (ratio of 3.8:1), this tendency is also found in other epidemiological studies, with a male- to female ratio varying between 1.8:1 to 5.4:1 [1, 3–5, 7, 9, 13, 15–19]. This variation could be explained by the more aggressive and violent nature of activities in which boys are taking part as compared to girls [15, 17, 20]. Niiranen et al. observed that accidents among girls occurred equally at all ages [21]. In our study, the risk of eye injury in girls was significantly higher in the youngest age group (< 7 years), while it increased with increasing age in boys and was highest in the oldest age group (13–18 years) (*p* < 0.001). This finding is found to be similar in studies published by Hosseini et al. from Iran [22], Ilhan et al. from Turkey [23] and Lee et al. from Taiwan [5]. The difference between genders increases considerably in the older age groups [21, 22]. The relatively lower ratio of males in the younger ages could be explained by the fact that at younger ages both boys and girls engage in similar daily activities [23].

Several studies state that injuries occur most frequently at home [4, 6, 7, 9, 18, 23, 24], emphasizing the importance of preventive measures in the home environment [6]. According to Ilhan et al. accidents in the streets and school were second in terms of frequency [23]. Similarly, our study found the majority of eye injuries to occur at home (60.4%), followed by outdoor settings such as yard or street (31.7%) and school (5.2%). In contrast, El-Sebity et al. [1] noted the road as a major place of injury in Egypt (54.7%) which is consistent with the study done by Sofi et al. [13] and Shoja et al. [12].

The place of injury is reported to vary according to age [23].Indeed, home was the most common place of injury in the < 7 age group in our study, whereas outdoor activities and street predominated in the 13–18 years age group. These findings are consistent with the studies performed by MacEwen et al. [9], Al-Mahdi et al. [4] and Ilhan et al. [23]. Younger children usually spend more time at home than older children [9], who are more likely to be engaged in unsupervised outdoor activities [4]. Our study showed that 5.2% of all eye injuries occurred at school. Some investigators report higher frequency of ocular trauma occurring in school settings: 31.33% for Desai et al. [24] and 12.3% for Al-Mahdi et al. [4]. This high incidence of trauma should encourage to pay more attention to safer environment for children at school.

In our study, blunt and sharp objects were the most common cause of eye injury, followed by burns, falls, explosions, gunshots, fireworks and traffic accidents. These results are consistent with data published by Serrano et al. [7]. Sharp object such as scissors and knives are usually found in every household. Sharp pieces of wood, toys and teeth/nails of animals significantly predominated in the pre-school age group and these data are consistent with studies from Turkey [23] and Malaysia [14]. In our study the most common reported blunt object was a wooden stick (24.8%), followed by sports equipment, stone and fruits or vegetables.

Significant preponderance of blunt objects and explosions among all causes of injury were observed in the age group of 7–12 years; and burns and fireworks – in children over 13 years of age. Analysis of the variety of blunt objects showed that children at the age of 7–12 were significantly more likely to suffer from projection of objects of various organic origin (potatoes, apples etc.), followed by stones and sticks, while fist and sports equipment were more likely to cause eye injury in older children. Saxena et al. [15] reported bow and arrow to be the most common cause of injury, followed by household appliances and firecrackers, which were the leading cause of ocular trauma among children in India. Wood, followed by stone and sharp objects were presented as the most common causes of eye injury in children by Sofi et al. [13]. Other studies conducted in African countries stated that 25% of ocular injuries in children were from gunshots, 24.2% from tools, and 21.8% from assault which reflects the cultural and socio-economical differences between countries [24]. According to Hosseini et al. [22] the high number of injuries caused by the use of sharp objects as toys remain a common problem in developing countries. Toys are a common cause of injury in the younger age group because of their inappropriate use (projection...). This should be considered and taken into account when establishing safety standards in manufacturing toys [9].

In our study CGI was the most common type of eye injury (53.4%), followed by OGI (28.7%), ocular burn and non-globe injury with significant differences in study age groups: the higher rate of CGI in children aged 13–18 years, and OGI in the pre-school age group. Results from the literature seem to corroborate our findings of a higher frequency of CGI [4–7, 9, 14, 16, 17, 20]. Different from those studies, other investigators observed a greater hospitalization rate in cases of OGI [1, 12, 15, 18, 22], which doesn’t imply that OGI are more frequent than CGI. Children with CGI are usually treated in an outpatient department and only the most severe cases (representing a small fraction of this category) required to be hospitalized. [22].

Analysis of OGI in our study showed that penetrating wounds were the most common type of open globe injury (74%) which is consistent with results from other studies [22, 25]. The rate of globe ruptures in our study was considerably lower (2.6%) than the 12% presented by Kadappu et al. [26], but the rate of IOFB injuries was higher in contrast to findings of Maurya et al. [18] and Kadappu et al. [26], who respectively reported only 4.88% and 4% of such injuries.

The majority (73.7%) of wounds were in zone I which is comparable to published results [22, 23, 26, 27]. Corneal laceration, hypotony, traumatic cataract, iris laceration/prolapse and vitreous prolapse were the most common presentations of OGI, whereas hyphema, secondary glaucoma and retinal edema were significantly related with CGI. El-Sebaity et al. [1] concluded, that in OGI corneal wounds predominated, while in CGI most cases presented with hyphema.

In our study 65.4% of injured children regained a good visual acuity (≥0.5). Comparisons with other studies are complicated due to the differences in study design and the great variability in the nature and severity of eye injuries themselves. Other pediatric studies reported achieving 6/12 or better in 36% [25], 74.5% [4] 88% [9], 92.7% [14], 68% [26], 75% [17], 46.34% [18] and 33% [23] of patients. In our study, 18.4% of patients reached a final VA of 0.1 or less, but here again, a great disparity of results is observed as Maurya et al. reported 19.51% (3/60) [18], Thompson et al. 15.3% (< 0.1) [25], Behbehani et al. 13% (< 20/400) [8], Ilhan et al. 33.3% [23], El-Sebaity et al. 86.6% (< 20/200) [1].

The final VA was related to the type and the severity of injury. We identified the OGI to be the most severe with a significantly high incidence of bad VA (grade 4 and grade 5) at the time of presentation. CGI were significantly related with an initial good VA (grade 1 and grade 2). Most CGI in our study (81.7%) did not cause any final visual impairment in the affected eye, whereas 32.8% of OGI caused severe visual impairment or blindness (VA ≤ 0.1). Other studies also concluded that OGI carry a poorer prognosis and are more likely to suffer from long-term visual impairment [1, 4–7, 15, 18, 20, 22, 28, 29].

OGI, in general, carry poorer prognoses and are more likely to require surgical intervention [18]. According to the literature, surgical intervention was required for 35% [17], 47.56% [18], 48% [9], 22.8% [14], 52.8% [4] of patients with OGI. In our study a surgery was performed in 42.13% of cases overall, and almost all OGI required surgery. All additional surgeries were required only in OGI.

We understand the limitations of our study, first of all due to the retrospective nature of our analysis which contained a large number of unrecorded data, and secondly related to the difficult management of pediatric patients: questionable circumstances surrounding eye trauma, difficulties in collecting data from ophthalmic examination (VA), variable follow up times, different surgeons etc.

The visual development of children continues until the age of 9–10 years, and despite a successful trauma management, amblyopia should be aggressively prevented in the early post-traumatic period as it could lead to a worse decrease of visual function than the injury itself [19]. Despite adequate therapeutic measures, the visual prognosis is still worse in children than in adults due to the nature of the injuries and the development of amblyopia [20]. Eye trauma in children can be prevented at several levels, firstly by proper children education to avoid potentially dangerous activities, and secondly at the parents, teachers and care-givers level by preparing safe environment (home, school…) for children.

## Conclusion

This study presents the characteristics of all the serious pediatric ocular trauma reported in the largest tertiary ophthalmological center of Lithuania from January 2008 until December 2013. The results of this study suggest the necessity to focus on developing new methods and strategies to aim at reducing the frequency of pediatric ocular trauma as a priority. Children suffer from a very wide range of ocular injuries and their visual outcome depends on the severity, the type of injury, as well as on the early management, extended follow up and prevention of amblyopia. In spite of improving healthcare and treatment possibilities, in our study, nearly one fifth of children had a very poor visual outcome after their injury.

## Additional file

Additional file 1: Complete data set including all the data collected from the patients' records. (XLS 409 kb)
